# The X-Rays in Sarcoma

**Published:** 1902-10-11

**Authors:** 


### THE X-RAYS IN SARCOMA.

Dr. William B. Coley [1] says that while the Roentgen rays have been used for some time in the treatment of superficial epithelial tumours very few data are available as to their efficacy upon sarcomatous tumours; but he now publishes a series of fourteen cases which have been so treated, and from the details gathered therefrom he concludes that the rays have a remarkable inhibitory action upon the growth of all forms of malignant disease; that this is especially true of sarcoma; and that in many cases of even far advanced and inoperable malignant disease this action may result in the total disappearance of the tumours, often without any breaking down of the tissues, the new growth being apparently absorbed. Whether the patients have been cured or the disease has merely been arrested to reappear at some future date, is a question that time alone can decide, but in any case it seems that the Roentgen ray has a very marked influence upon the pain of nearly all types of malignant tumours, causing entire relief in the majority of cases. It should be noted that in some instances the patients were in addition treated with Coley's mixed toxins, and also, which is very curious, that in one case, at least, coincidently with the reduction of the tumour which was being X-rayed, other tumours which had not been exposed to the rays also diminished in size.

[1] New York Medical News, September 20.

